# Landscape of copy number aberrations in esophageal squamous cell carcinoma from a high endemic region of South Africa

**DOI:** 10.1186/s12885-020-06788-3

**Published:** 2020-04-06

**Authors:** Jacqueline Brown, Andrzej J. Stepien, Pascale Willem

**Affiliations:** 1 School of Pathology, Department of Molecular Medicine and Haematology, Faculty of Health Sciences, University of the Witwatersrand, Johannesburg and the National Health Laboratory Services, Johannesburg, South Africa; 2 Department of Anatomical Pathology, School of Medicine, Faculty of Health Science, Walter Sisulu University, National Health Laboratory Services/NMAH, Mthatha, South Africa

**Keywords:** Esophageal, Squamous, Carcinoma, Copy number, Microarray

## Abstract

**Background:**

Esophageal squamous cell carcinoma (ESCC) is an aggressive cancer with one of the highest world incidences in the Eastern Cape region of South Africa. Several genome wide studies have been performed on ESCC cohorts from Asian countries, North America, Malawi and other parts of the world but none have been conducted on ESCC tumors from South Africa to date, where the molecular pathology and etiology of this disease remains unclear. We report here tumor associated copy number changes observed in 51 ESCC patients’ samples from the Eastern Cape province of South Africa.

**Methods:**

We extracted tumor DNA from 51 archived ESCC specimens and interrogated tumor associated DNA copy number changes using Affymetrix® 500 K SNP array technology. The Genomic Identification of Significant Targets in Cancer (GISTIC 2.0) algorithm was applied to identify significant focal regions of gains and losses. Gains of the top recurrent cancer genes were validated by fluorescence in situ hybridization and their protein expression assessed by immunohistochemistry.

**Results:**

Twenty-three significant focal gains were identified across samples. Gains involving the *CCND1, MYC, EGFR* and *JAG1* loci recapitulated those described in studies on Asian and Malawian cohorts. The two most significant gains involved the chromosomal sub-bands 3q28, encompassing the *TPRG1* gene and 11q13.3 including the *CTTN*, *PPFIA1*and *SHANK2* genes*.* There was no significant homozygous loss and the most recurrent hemizygous deletion involved the *B3GAT1* gene on chromosome 11q25. Focal gains on 11q13.3 in 37% of cases (19/51), consistently involved *CTTN* and *SHANK2* genes. Twelve of these cases (23,5%), had a broader region of gain that also included the *CCND1*, *FGF19, FGF4* and *FGF3* genes. *SHANK2* and *CTTN* are co-amplified in several cancers, these proteins interact functionally together and are involved in cell motility. Immunohistochemistry confirmed both Shank2 (79%) and cortactin (69%) protein overexpression in samples with gains of these genes. In contrast, cyclin D1 (65%) was moderately expressed in samples with *CCND1* DNA gain.

**Conclusions:**

This study reports copy number changes in a South African ESCC cohort and highlights similarities and differences with cohorts from Asia and Malawi. Our results strongly suggest a role for *CTTN* and *SHANK2* in the pathogenesis of ESCC in South Africa.

## Background

Esophageal squamous cell carcinoma (ESCC) is an aggressive cancer which occurs in specific regions of the world which include Lixian China, Japan, the Golestan province of Iran, parts of South America (Uruguay) and the eastern corridor of Africa, (Malawi, Kenya and South Africa (SA) [1–3]. In South Africa, the Eastern Cape province has one of the highest world incidences of 31.3 and 18 per 100,000 male and female individuals respectively [4]. A number of early studies in western countries have identified ESCC risk factors such as alcohol consumption and smoking. However, these risk factors are absent in a number of high endemic areas where other causes, including nutritional deficiencies, lower socio-economic status, consumption of hot beverages and exposure to polycyclic aromatic hydrocarbons are suspected [2, 3]. ESCC risk has also been related to the consumption of maize contaminated by aflatoxin [5, 6] and in South Africa, chronic inflammation caused by a local cultural practice of induced vomiting, was thought to play a role [7]. The respective impact of these factors is unclear and environmental/cultural exposures are likely to interact with population specific genetic susceptibilities. The dismal prognosis of this disease [third cause of death in SA [8], and first cause of death in both males and females in the Eastern Cape region (unpublished data from community-based cancer registry)] underscores the need to understand its molecular pathology.

Several genome-wide copy number studies have been performed on ESCC cohorts from Asian and western countries, using technologies of varied resolutions. The most recurrent somatic copy number variations (SCNV) across these studies involve gains on chromosomes 3q26-q29, 7p11.2-p22.1, 8q22.3–24.21, 11q12.3-q13.4 and 20q11-q13.33 and losses on chromosomes 3p11.1–14.2, 8p21.3-p23.2, 9p21.3–24.1 and 18q11-q22.3. These regions host key cancer genes including *PIK3CA*, *SOX2*, *EGFR*, *MYC, CCND1*, *CTTN*, *FHIT* and *CDKN2A*/*B* [9–14]. The most common recurrent gains across studies involves the 11q12.3–13.4 region with amplicons of varied size that almost always include the oncogene *CCND1* [9–15]. Apart from copy number aberrations, mutational analyses have shown recurrent inactivating mutations in *TP53*, and *NOTCH1* as well as activating events in *PIK3CA* [10, 11, 15]. A single genomic study, performed on African patients from Malawi, recapitulated patterns of gene mutations and copy number changes (gains of *CCND1*, *TP63, MYC, ERBB2, EGFR, MYCL1* and losses of *CDKN2A/CDKN2B),* similar to those observed in Asian and North American ESCC patients [16]*.* Of note, gene expression patterns from transcriptome sequence analysis in this African cohort highlighted three distinct ESCC subgroups that tended to reflect exposure to differing environmental factors [16]. The diversity in the genomic landscape observed in this study strongly warrants the expansion of genomic investigations in other African countries with high ESCC incidence in order to infer etiologic factors and identify markers of disease with a potential for early detection and improved therapeutic interventions.

Apart from a report using conventional cytogenetic comparative genomic hybridization (CGH) [17], and a study on five ESCC cell-lines established in SA [18], there are no high-resolution genome wide SCNV data on ESCC in South Africa. We report SCNVs in 51 ESCC tumor specimens derived from a single geographic region of South Africa that shows one of the highest world incidences for this disease.

## Methods

### Tumor material and patient characteristics

Eighty-two archived, formalin fixed paraffin embedded (FFPE) ESCC specimens were collected from the archives of the Nelson Mandela Academic Hospital in Mthatha, Eastern Cape from the years 2004–2006. The ratio of males to females was 1:1.16. Haematoxylin and eosin stained slides were reviewed and marked by an experienced pathologist to identify tumor areas (> 80% tumor cells) for DNA extraction. Thirty FFPE samples with a normal tissue histology from a matched population (age and ethnicity) were collected from the same laboratory and constituted the reference panel for copy number analysis.

### Genomic DNA isolation

Tumors and control specimens were pre-treated in 1 M sodium thiocyanate and DNA was extracted using proteinase K digestion followed by phenol/chloroform extraction. DNA quality was assessed by standard gel electrophoresis and spectrophotometry. FFPE DNA is known to show varying degrees of degradation and to establish the ability of these samples to amplify large fragments, a multiplex PCR assay (previously described) was performed prior to array processing [19]. Of 82 ESCC samples collected, 51 yielded enough quality DNA to proceed with SNP arrays.

### Affymetrix 500 K SNP array

DNA from ESCC and control specimens were hybridized to Affymetrix® 250 K Nsp and Sty GeneChips® respectively, which have a mean probe spacing of 5.8 kb. Samples were hybridized once per chip type. The Affymetrix® GeneChip® mapping 500 K protocol (P/N 701930 Rev. 3) was followed, apart from the number of PCR reactions per sample, which was increased to six to yield the optimal amount of 90 μg of PCR product. Scanning was performed on the Affymetrix® GeneChip Scanner 3000 7G (Affymetrix®, Santa Clara USA). The data discussed in this publication have been deposited in NCBI’s Gene Expression Omnibus [20] and are accessible through GEO Series accession number GSE59105 (http://www.ncbi.nlm.nih.gov/geo/query/acc.cgi?acc=GSE59105).

### 500 K data analysis

Raw intensity data (CEL files) were imported into Genotyping Console™ (Affymetrix®, Santa Clara USA) to assess the SNP call rates as an initial quality control measure. The average call rates were 71.3 and 72.1% for Nsp and Sty respectively. Call rates were expected to be lower than for fresh tissue (93–95%) due to poor amplification of larger fragments during PCR [21]. The raw intensity data of 50 samples were imported into Partek® Genomics Suite where quantile normalization, SNPs on fragments larger than 700 bp were removed and copy number analysis were performed. The copy number data were segmented using the circular binary algorithm in GenePattern [22] using a minimum of 10 markers for regions of gain and loss. Common copy number variants were removed from the data after comparing each region of change to the Database of Genomic Variants (http://projects.tcga.ca/variation). To assess the significance of gains and losses, the segmentation file was analysed using GISTIC 2.0 ^ref^ (Genomic Identification of Significant Targets in Cancer) [23] using a q-value cut-off of 0.25.

Common regions of gain or loss and the respective genes involved were reported using the Refseq database, genome build hg18.

### Fluorescence in situ hybridisation (FISH)

Gains of *CCND1*, and *MYC* were validated on 10 samples using the LSI t(11;14) dual color dual translocation probe (Abbott Molecular, USA), which covers the *CCND1* and *FGF4* loci on chromosome 11 and the LSI MYC SpectrumOrange probe (Abbott Molecular, USA) respectively. BAC clones were obtained from the BACPAC resource center, Children’s Hospital Oakland Research Institute, CA, USA. The BAC clone, RP11-736 L3 (Chr 11: 70,732,999-70,899,011), mapping to *SHANK2* gene on 11q13.3 was labeled by nick translation with SpectrumOrange-dUTP (Abbott Molecular, USA) and hybridized to 10 ESCC samples as described previously [18]. Briefly, three-micron sections were baked at 60 °C overnight and de-waxed twice in Xylene (Merck). Dehydrated slides were pre-treated in 0.2 N HCl for 20 min, followed by 1 M sodium thiocyanate at 80 °C for 30 min. Air dried slides were treated with Pepsin (Roche) (0.5 mg/ml) for 20 min to 1 h30 minutes at 37 °C depending on the tissue size and thickness. Slides were rinsed in 2x SSC, dried at 42 °C and fixed in 1% formaldehyde at room temperature. Pre-treated samples were denatured in 50% formamide buffer at 76 °C for 5 min, dehydrated in ice-cold ethanol and denatured probes (76 °C for 5 min) were added for overnight hybridisation at 37 °C. The next day, slides were washed in 2x SSC at 76 °C for 5 min, counterstained with DAPI and mounted using Vectashield® fluorescent mounting medium (Vectalabs, USA). Images were captured using Cytovision 4.0 (Applied Imaging) on an Olympus BX61 fluorescent microscope.

### Immunohistochemistry (IHC)

In order to assess the protein expression of the most recurrent target genes, we performed immunohistochemistry on 4 μm deparaffinised sections in duplicate. The DAKO EnVision FLEX detection system was used according to the manufacturer’s instructions. Cyclin D1 was detected using ready-to use FLEX monoclonal anti-cyclin D1 (Clone EP12, Dako IR08361) as supplied. The Cortactin and Shank2 proteins were detected using rabbit monoclonal anti-cortactin antibody (EP1922Y, Abcam, 0.095 mg/ml) diluted to 1:250 and rabbit polyclonal anti-Shank2 antibody (aa 331–380, Abcam, 1 mg/ml) diluted to 1:75 respectively. Slides were counterstained with Haematoxylin and mounted in aqueous mounting solution. Positive controls were respectively, breast tumour for Cortactin, mantle cell lymphoma for Cyclin D1 and staining observed in suprabasal epithelial cells of normal oesophageal squamous epithelium for Shank2. The primary antibody was replaced with antibody diluent as a negative control. To correlate the gains of *SHANK2*, *CCND1* and *CTTN* genes with their respective protein expression, samples with gains of these 3 genes (*n* = 22), gains of *SHANK2* alone (n = 2) and no gains (n = 2) were processed. Staining was scored on the intensity (0–3) and the percentage of positive cells (0 = no staining, 1 = < 10% with moderate staining, 2= > 10% with moderate staining and 3 ≥ 50% with intense staining.

## Results

**Fig. 1:**
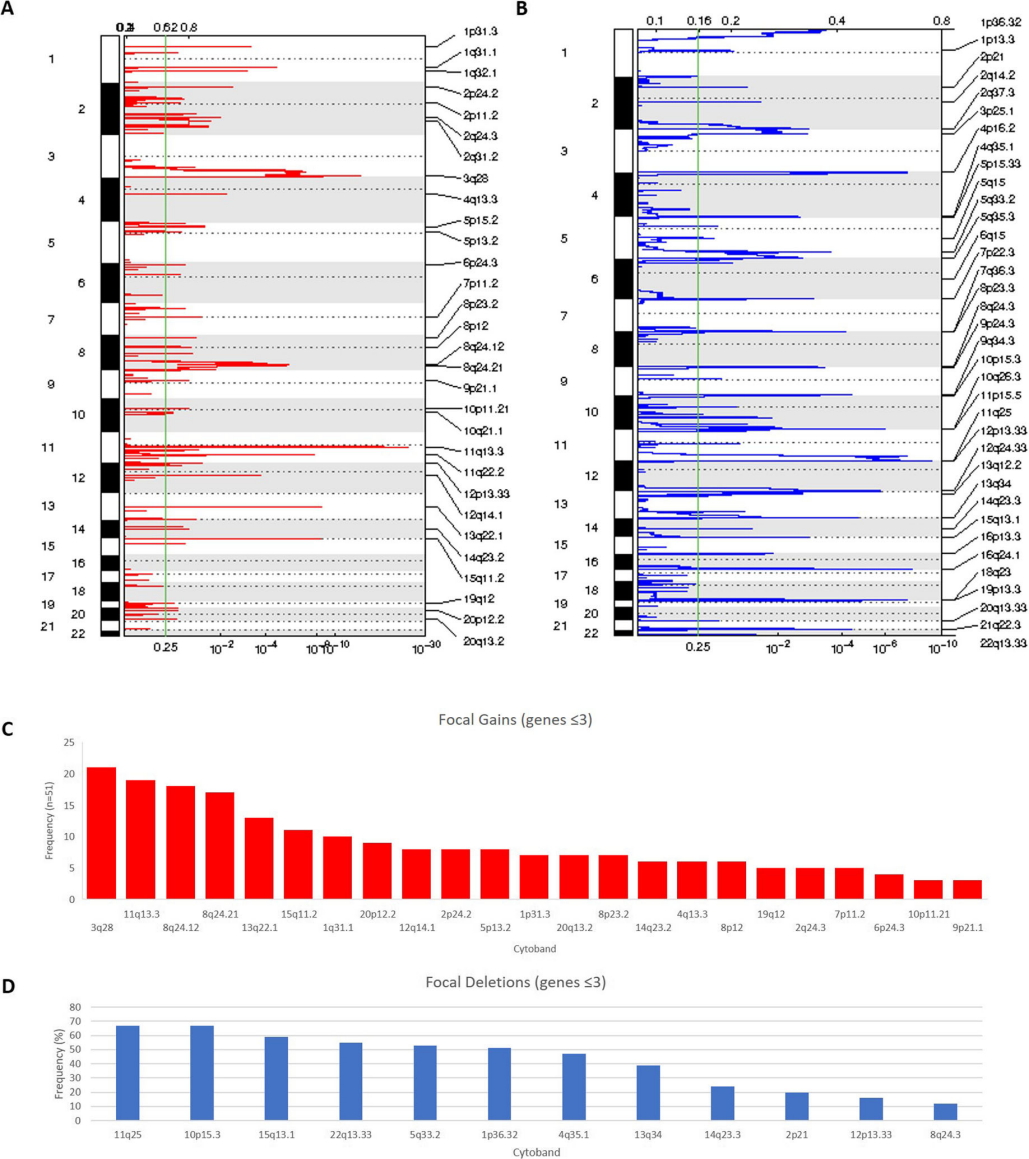
Summary of gains and loss identified by GISTIC 2.0. **a** Copy number gains identified in ESCC by GISTIC 2.0**. b** Copy number deletions detected by GISTIC 2.0**. c** Graph representing focal gains (≤3 genes) identified by GISTIC 2.0 analysis sorted by frequency. **d** Graph showing focal deletions (≤3 genes) detected by GISTIC 2.0 analysis sorted by frequency

Array copy number analysis of South African ESCC samples revealed a high level of complexity in the tumor genome with most chromosomes showing aberrations, (median number of aberrations per case: 96, minimum: 33, maximum: 426). GISTIC 2.0 analysis identified 30 gains (Supplementary Table 1) and 36 deletions (Supplementary Table 2) (Fig. 1a and b)**.**

### Gains

Twenty-three focal gains (≤3 genes) were observed (Table 1). Those involved chromosomes 1q31.1, 1p31.3, 2p24.2, 2q24.3, 3q28, 4q13.3, 5p13.2, 6p24.3, 7p11.2, 8p12, 8p23.2, 8q24.12, 8q24.21, 9p21.1, 10p11.21, 11q13.3, 12q14.1, 13q22.1, 14q23.2, 15q11.2, 19q12, 20p12.2 and 20q13.2. The two top recurrent gains involved

**Table 1:** Focal gains identified by GISTIC 2.0 analysis (regions with ≤3 genes). Regions are ordered by chromosome

Cytoband	***q*** value	Peak boundaries	Approximate Size (kb)	Frequency (***n*** = 51) (%)	Genes
1q31.1	2.3726e-05	chr1:185468920–185,520,599	51,679	10 (19.6)	*PLA2G4A*
1p31.3	0.00062772	chr1:66762738–66,812,099	49,361	7 (13.7)	*SGIP1*
2p24.2	0.0036518	chr2:17635668–17,792,214	156,546	8 (15.7)	*VSNL1, SMC6*
2q24.3	0.010142	chr2:165491226–165,903,111	411,885	5 (9.8)	*SCN2A, SCN3A, SLC38A11*
3q28	1.9145e-14	chr3:190233839–190,297,244	63,405	21 (41.2)	*TPRG1*
4q13.3	0.0063346	chr4:74554931–74,770,220	215,289	6 (11.8)	*AFM, RASSF6*
5p13.2	0.12506	chr5:36212218–36,345,590	133,372	8 (15.7)	*SKP2, C5orf33, RANBP3L*
6p24.3	0.10455	chr6:7469233–7,587,193	117,96	4 (7.8)	*DSP, C6orf151*
7p11.2	0.039529	chr7:54888060–55,205,929	317,869	5 (9.8)	*EGFR*
8p12	0.072453	chr8:36981731–37,716,301	734,57	6 (11.7)	*ERLIN2, ZNF703*
8p23.2	0.05569	chr8:4993944–5,001,641	7697	7 (13.7)	*CSMD1*
8q24.12	3.026e-06	chr8:122208528–122,239,169	30,641	18 (35.3)	*SNTB1*
8q24.21	4.72e-06	chr8:128624619–128,707,294	82,675	17 (33)	*MYC*
9p21.1	0.082133	chr9:31568898–31,803,849	234,951	3 (5.9)	*ACO1*
10p11.21	0.080762	chr10:35074847–35,469,974	395,127	3 (5.9)	*CREM, CUL2, PARD3*
11q13.3	2.782e-25	chr11:69889604–70,002,885	113,281	19 (37.3)	*CTTN, PPFIA1, SHANK2*
12q14.1	0.00019309	chr12:59418827–59,513,190	94,363	8 (15.7)	*FAM19A2*
13q22.1	3.1551e-09	chr13:73904231–74,055,232	151,001	13 (25.5)	*KLF12*
14q23.2	0.080762	chr14:61922478–62,321,423	398,945	6 (11.8)	*KCNH5*
15q11.2	5.1116e-09	chr15:22380933–22,441,820	60,887	11 (21.6)	*C15orf2*
19q12	0.17889	chr19:30530936–30,776,391	245,455	5 (9.8)	*[UQCRFS1]*
20p12.2	0.14276	chr20:10451892–11,654,335	1202,443	9 (17.6)	*JAG1, C20orf94*
20q13.2	0.15938	chr20:52721957–52,854,653	132,696	7 (13.7)	*DOK5*

the *TPRG1* gene on 3q28 (21/51 cases, 41%), and the *CTTN, PPFIA1* and *SHANK2* genes on 11q13.3 (19/51, 37%) (Fig. 1c)**.** Although the function of the *TPRG1* gene is not well established, amplification and/or activating mutations in Cis regulatory elements of this gene associated with its increased expression have recently been reported in diffuse large B-cell lymphomas, suggesting potential oncogenic activity [24].

Chromosome 11q13.3 gain is a common event in ESCC, where it almost always involves the *CCND1* proto-oncogene [9–11, 13] and, to a lesser extent, the *CTTN* and *SHANK2* genes. In our cohort *CTTN* and *SHANK2* were the most frequent amplified genes at 11q13.3 and this region expanded proximally to include the *CCND1, FGF19, FGF4* and *FGF3* in 12 / 51 cases.

The cortactin protein, encoded by the *CTTN* gene, is an actin binding scaffolding protein with various cellular functions and is known to promote cell motility [25]. The Shank2 protein belongs to another family of scaffolding proteins and is a cortactin binding partner [26]. It has mostly been studied in neuronal synapses and its role in cancer is unclear [27]. Similarly, the *PPFIA1* gene, which encodes the cytosolic scaffolding protein lyprin-α1 [28], is a potential target gene often co-amplified at 11q13.3 with *CCND1* and the above two genes in ESCC [29].

*CCND1* encodes a protein which promotes cell cycle progression. Gain thereof and associated increased expression is well described in a variety of cancer types including head and neck squamous cell carcinoma and ESCC [13–16, 30].

Other notable significant focal gains involved the known proto-oncogenes *EGFR* and *MYC* on 7p11.2 and 8q24.21 respectively (Table 1). *EGFR* copy gains are seen in approximately 20% of ESCC patients, who show improved survival when treated with the anti-EGFR kinase inhibitor, gefitinib [31].

**Fig. 2:**
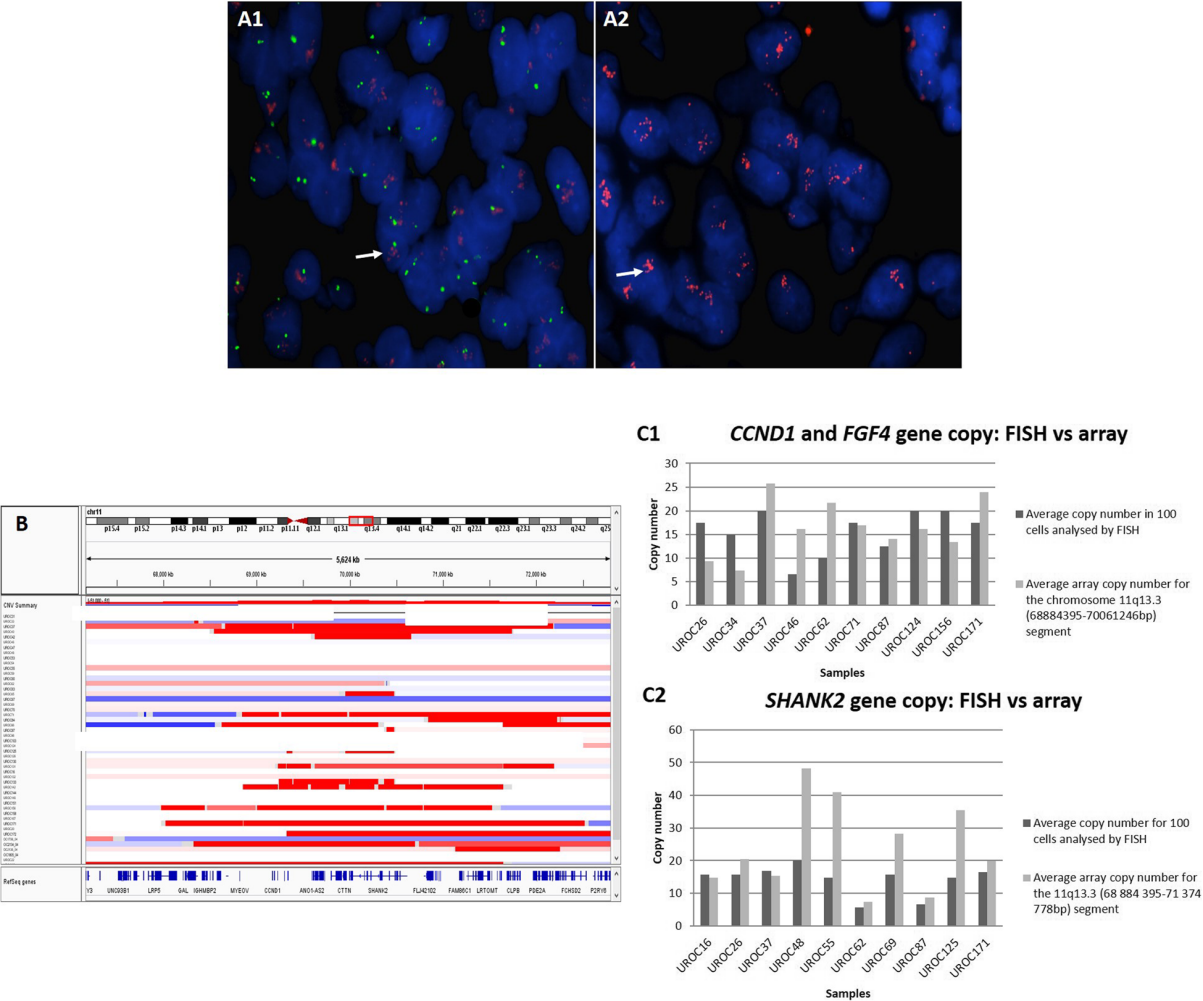
*CCND1*/*FGF4* and *SHANK2* genes copy number. (A) DAPI stained nuclei from sample UROC171. **a**1 FISH analysis was performed with the Vysis LSI t(11;14) dual color probe. The *IGH* gene probe on chromosome 14, acts as an internal control (green signal), the red signal represents locus specific probe encompassing the *CCND1* and *FGF4* genes). Gains are seen with 6–8 red signals (white arrow) while the control probe shows two green signals in most cells. **a**2 DAPI stained nuclei from UROC171 case, hybridized with the BAC clone, RP11-736 L3 (*SHANK2* gene), labeled with SpectrumOrange-dUTP (Abbott Molecular, USA). Clumping of red signals for *SHANK2* (white arrow), were consistent with high-level gains. This type of signal pattern was approximated to 20 signals. **b** 500 K SNP copy number segmentation for chromosome 11q in all samples generated in GenePattern (IGV). The minimal common region of gain (11q13.3: 69889604–70,002,885) is represented by the red box. This region includes the *CTTN, PPFIA1* and *SHANK2* genes. **c** Graphs showing the average copy number of *CCND1* and *SHANK2* for each of the 10 samples analyzed by FISH. **c**1 The average *CCND1* copy number across 10 samples was 15.7 by FISH and 16,5 by copy number array analysis (11q: 68884395-70,061,246 bp) in the same cases. **c**2 Gain of *SHANK2* was confirmed by FISH in 10 cases (average of 14,2 copies), the same cases had an average copy number of 23,5 by array copy number (11q:70,061,246-70,310,057)

FISH confirmed gains of *SHANK2* and *CCND1* in 10 cases and matched closely with array analysis data (Fig. 2).

### Evaluation of cyclin D1, Shank2 and cortactin proteins expression

**Fig. 3:**
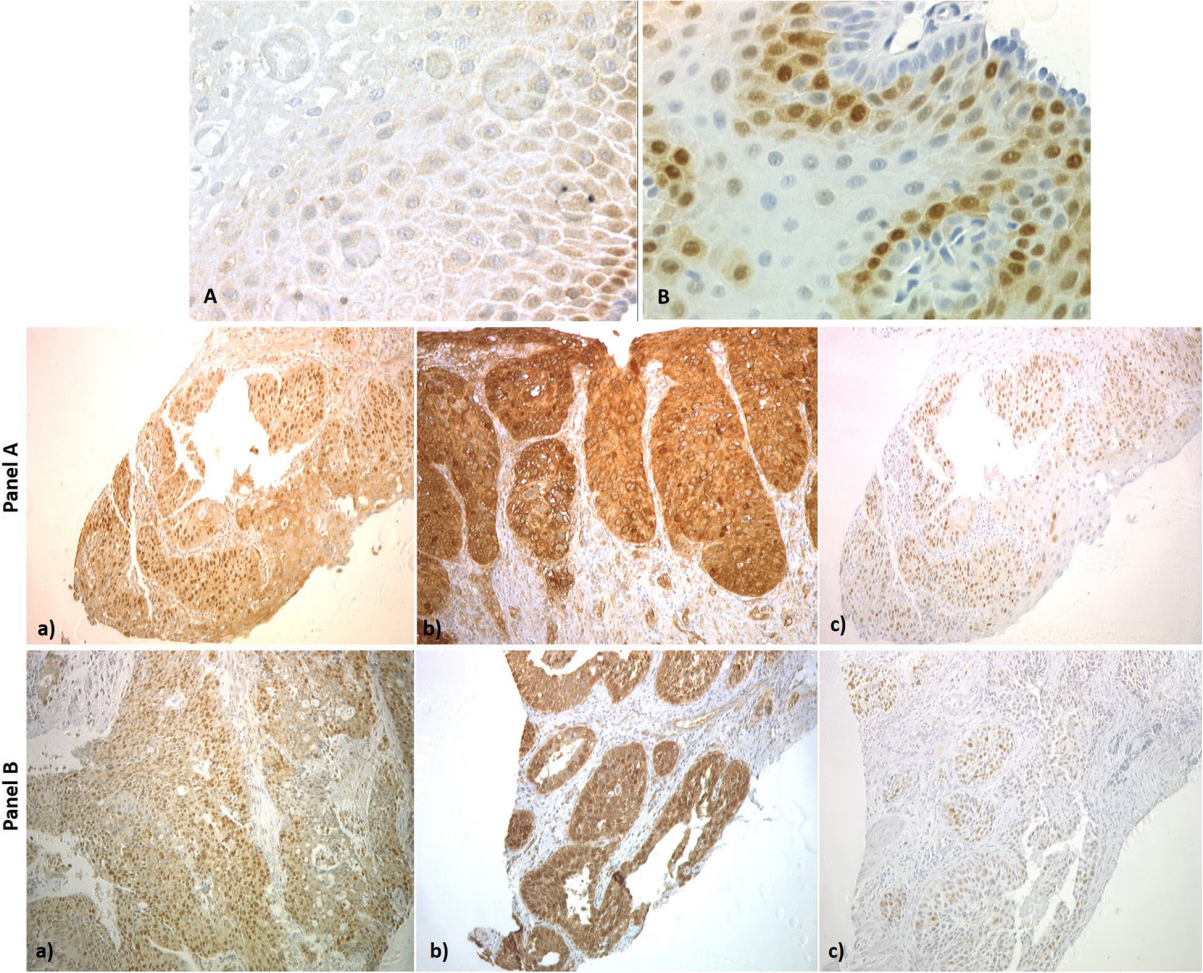
Representative images of the common immunohistochemical staining patterns for Shank2, cortactin and cyclin D1. **a** shows Shank2 staining (40x magnification) in non-neoplastic oesophageal squamous mucosa, cytoplasmic signal was observed in basal cells, which disappeared towards the luminal surface. **b** shows staining of *CCND1* in non-neoplastic oesophageal squamous mucosa (40x magnification), staining was observed in nuclei, which disappeared towards the luminal surface. **Panel A:** Case UROC48 with co-amplification of the *SHANK2*, *CTTN* and *CCND1* genes. **a)** shows intense cytoplasmic staining for Shank2 (score 3). **b)** intense cytoplasmic and membranous staining for cortactin (score 3). **c)** Moderate staining for cyclin D1 (score 1). **Panel B**: Case UROC144 with amplification of the *SHANK2* gene only. **a)** shows intense cytoplasmic staining for Shank2 (score 3), **b)** shows intense cytoplasmic staining for cortactin (score 3) and **c)** shows moderate staining for cyclin D1 (score 2)

To assess if the most common gains resulted in increased protein expression of target genes, we assessed Shank2 and cortactin immunoreactivity in normal and tumor esophageal tissues. Signals for both proteins were low in non-neoplastic esophageal squamous epithelium, in the cytoplasm (Shank2) or nuclei (cortactin), of basal epithelial cells, and disappeared in cells leaning towards the luminal surface (Fig. 3). Twenty-six tumor samples were assessed for Shank2, cortactin and cyclin D1 protein expression; of these, 22 cases had DNA gain of all three genes and 19/22 (86%) overexpressed Shank2 (score3), 16/22 (72%) overexpressed cortactin, while only 5/22 cases (22%) overexpressed cyclin D1, (score of 3). Cyclin D1 was moderately expressed in 12/22 cases (54%) (score of 2) (Fig. 3, panel a). Overall, 19/26 (73%) and 18/26 (69%) of cases overexpressed Shank2 and cortactin respectively. One case had gain of *CCND1* only, but all three genes showed moderate protein expression on IHC. One sample with *SHANK2* gain only, overexpressed Shank2 as well as cortactin, while cyclin D1 was moderately expressed (Fig. 3, Panel b). One case had no gains of these three genes and over expressed cortactin, while Shank2 and cyclin D1 were weakly expressed (score 1). In summary, Shank2 and cortactin were co-expressed in most cases with gains of these genes. Co-amplification of *CTTN*, *SHANK2* and *CCND1* genes has been reported previously in oral squamous cell carcinoma. In contrast to our study all cases overexpressed cyclin D1 (quantitative PCR analysis), while a subset of cases 50% overexpressed *CTTN* and *SHANK2* [32].

### Losses

Twelve significant focal deletions were detected by GISTIC 2.0 analysis (Table 2 and Fig. 1d). All losses were heterozygous. These deletions covered chromosomal regions 1p36.32, 2p21, 4q35.1, 5q33.2, 8q24.3, 10p15.3, 11q25, 12p13.33, 13q34, 14q23.3, 15q13.1 and 22q13.33. The most frequent losses were on chromosome 11q25 (67%) and 10p15.3 (66%). Both regions covered one gene, *B3GAT1* and *ADARB2* respectively. *ADARB2* has no known role in cancer. *B3GAT1*, also known as *CD57*, expression was previously tested in 3672 prostate cancer and benign specimens by IHC. While *CD57* was expressed in benign prostate and low-grade prostate cancer, loss of expression correlated with tumor de-differentiation and size [33]. Three other regions of loss harbored genes with a known tumor suppressor function. These included the *ZFP36L2* gene on 2p21, *ING2* on 4q23.3 as well as the microRNA *MIR625,* and *FUT8* gene on 14q23.3. *ZFP36L2* is a putative transcription factor involved in cellular responses, which was shown to act as a tumor suppressor in colorectal cancer and acute myeloid leukemia [34, 35]. Lack of expression of the known tumor suppressor *ING2,* a chromatin remodeling protein*,* has been reported in several types of cancer [reviewed in [36]]. Decreased expression of *MIR625* was described in colorectal carcinoma. Expression of this microRNA in colorectal metastatic models in nude mice was shown to suppress cell invasion and metastasis suggesting a tumor suppressor activity [37]. Decreased expression of *MIR625* was reported in ESCC patients previously where it was associated with a 5-year decreased survival rate (38.1%) compared to ESCC patients with higher *MIR625* expression [38].

**Table 2:** Focal Deletions identified by GISTIC 2.0 analysis. Regions are ordered by chromosome

cytoband	***q*** value	wide peak boundaries	Size (kb)	Frequency (***n*** = 51) (%)	Gene
1p36.32	4.4821e-06	chr1:2546230–3,101,761	555,531	26 (51)	*ACTRT2*
2p21	0.043569	chr2:42871145–43,761,298	890,153	10 (19.6)	*ZFP36L2, THADA, LOC728819*
4q35.1	0.0032739	chr4:184659448–185,070,554	411,106	24 (47)	*ING2, C4orf41, RWDD4A*
5q33.2	0.00026001	chr5:153410221–153,828,954	418,733	27 (53)	*GALNT10, SAP30L*
8q24.3	0.11261	chr8:140741552–141,656,154	914,602	6 (11.8)	*CHRAC1, NIBP*
10p15.3	0.000616	chr10:1166401–3,107,538	1941,137	34 (66.7)	*ADARB2, C10orf109*
11q25	7.4472e-10	chr11:133707909–134,452,384	744,475	34 (67)	*B3GAT1*
12p13.33	0.023341	chr12:417634–738,596	320,962	8 (15.7)	*NINJ2, B4GALNT3*
13q34	2.2677e-05	chr13:113562426–113,786,946	224,52	20 (39)	*FAM70B*
14q23.3	0.034764	chr14:64959313–66,072,039	1112,726	12 (23.5)	*hsa-mir-625, FUT8*
15q13.1	0.001437	chr15:25429109–26,306,775	877,666	30 (58.8)	*OCA2, HERC2*
22q13.33	4.0476e-05	chr22:49396414–49,482,863	86,449	28 (55)	*ARSA*

## Discussion

We determined the pattern of segmental gains and losses in ESCC tumors from South African patients of the Eastern Cape Province, a region with one of the highest ESCC incidences in the world, using high resolution 500 K SNP array technology. Our results showed both differences and similarities in SCNVs compared to studies performed on ESCC cohorts form Asia and Malawi. The high number (96 mean aberrations per case) and heterogeneous nature of SCNVs was in keeping with the notion that ESCC is a genetically complex disease [9–11, 13].

Large-scale gains on chromosomes 3q, 8q and 11q, observed in this study were similar to those reported previously [9–14]. One of the most frequent (88%) common focal regions of high copy gain on chromosome 11q13 observed here almost always involved the *CTTN*, *SHANK2* and *PPFIA1*genes.

The *SHANK2* and *CTTN* genes are in close proximity (30 kb) and are often co-amplified in oral squamous cell carcinoma [32]. These two genes’ protein products interact together and in its epithelial isoform, Shank2 binds to the SH3 domain of cortactin. Shank2-cortactin interaction was shown to facilitate cell motility by preventing anoikis through the PI3-Akt pathway in neural cells [27, 39]. One can hypothesise that such interaction may occur in ESCC thus facilitating cell motility and metastasis. *CTTN* gain/ increased expression alone has been associated with ESCC metastasis and functional studies further demonstrated that inhibition of *CTTN* expression decreased tumor growth and lung metastasis [27]. Additionally, two previous studies reported overexpression of *CTTN* in ESCC pre-cancerous lesions [40, 41]. In addition, in the 11q13.3 region of focal gain, the *PPFIA1* gene has not been studied extensively in ESCC but was shown to be significantly overexpressed in head and neck squamous cell carcinoma [42].

In our South African cohort, 12/51 cases had a broader region of gain on chromosome 11q13.3, which included the known oncogenes *CCND1*, *FGF3*, *FGF4, FGF19* as well as the recently described oncogenic *MIR548K* [10]. This broader region of gain has been described in a number of previous investigations including in 5 ESCC cell-lines established in South Africa [9–18]. In our cohort, cyclin D1 expression correlated to a lesser extent with gains of *CCND1* (5/23 cases) than Shank2 and cortactin. *CCND1* remains an important candidate in ESCC as a known oncogene involved in a number of malignancies and as a notable cell cycle regulator [13, 42]. *MIR548K*, shown to enhance cell proliferation in ESCC cell-lines [13], may also be a candidate key gene considering that this micro RNA lies within the broader region of gain on chromosome 11q13 in the present cohort.

The significant region of focal gain detected on chromosome 3q28, targeted the *TPRG1 (*tumor protein p63 regulated 1*)* gene. Although this gene has not been linked to ESCC pathogenesis, its distal neighbor gene, *TP63* showed gains in a wider peak region, in 20 of the 21 cases with gains at 3q28. *TP63* is a significant target of 3q gain in ESCC patients from Malawi as well as in ESCC cohorts from Western and Asian countries [16, 43]. Of note, *TPRG1* is highly expressed in normal esophageal tissue and an intergenic susceptibility locus (rs6791479) was identified in a genome-wide association study of cutaneous squamous cell carcinoma in between the *TP63* and *TPRG1* genes [44]. Taken together with the fact that the ESCC genomic profile is closer to other squamous cell carcinomas than to esophageal adenocarcinoma, the above observations support the notion that one or both these genes may play an important role in South African ESCC pathogenesis [43].

Chromosome 3q amplicons have been described across a number of ESCC studies and usually involve the *PIK3CA* and/or *SOX2* genes [9, 10, 12, 14]. By contrast to the cohort in Malawi, these genes did not show copy number alteration in our cases [16]. Mutational analysis would have to be performed to exclude activating mutations.

Significant gains involving the oncogene *MYC* were observed in our cohort, in keeping with studies that implicated the 8q24.1-q24.2 chromosomal region in other populations [9, 10, 13, 14, 16]. Similarly, gains involving the *EGFR* gene at chromosome 7p11.2 are previously described and thought to play a role in ESCC pathophysiology [9, 10, 13, 16, 18].

There were no significant homozygous deletions in this series as per GISTIC 2.0 analysis. Of note, no losses at the *CDKN2A, CDKN2B* and *TP53* loci were detected in this cohort in contrast with losses observed in the cohort from Malawi [16]. Although this could be due to incorrect array normalization, it is unlikely since our FISH results correlated tightly with arrays results.

We acknowledge limitations of this study due to the lack of patients’ clinical data and that aberrations detected could not be correlated with risk factors endemic to the region. No correlation could be established between copy number variants and stages of disease. Genome wide mutational analysis was also not performed in the present study and is currently being conducted on South African samples as part of a larger international collaboration.

## Conclusions

This study describes both common and differing regions of copy number aberrations in ESCC from South Africa when compared to other cohorts. Of note, our results suggest a role for Shank2 and cortactin proteins in ESCC carcinogenesis in South Africa. This will have to be clarified by future functional studies with a view to developing new markers of disease.

## Supplementary information

**Additional file 1.** Supplementary Table 1. Table of all gains detected by GISTIC 2.0. Supplementary Table 2. Table of deletions detected by GISTIC 2.0

## Data Availability

The datasets generated and/or analysed during the current study are available in NCBI’s Gene Expression Omnibus [20] and are accessible through GEO Series accession number GSE59105 (http://www.ncbi.nlm.nih.gov/geo/query/acc.cgi?acc=GSE59105).

## Abbreviations

BAC: Bacterial artificial chromosome
ESCC: Esophageal squamous carcinoma
FFPE: Formalin fixed paraffin embedded
FISH: Fluorescence in situ hybridisation
GEO: Gene Expression Omnibus
GISTIC: Genomic Identification of Significant Targets in Cancer
IHC: Immunohistochemistry
PCR: Polymerase chain reaction
SCNV: Somatic cop number variants
SNP: Single nucleotide polymorphism
SSC: Saline sodium citrate
